# Associations Between Elevated Rates of Depression, Anxiety, and PTSD Among ICU Survivors and Increased Mortality and Readmissions

**DOI:** 10.1002/brb3.70319

**Published:** 2025-02-16

**Authors:** Chen‐Shu Chang, Fuu‐Jen Tsai, Chun‐Hui Liao

**Affiliations:** ^1^ Department of Neurology, Vascular and Genomic Research Center Changhua Christian Hospital Changhua Taiwan; ^2^ Department of Medical Laboratory Science and Biotechnology Central‐Taiwan University of Science and Technology Taichung Taiwan; ^3^ School of Chinese Medicine, College of Chinese Medicine China Medical University Taichung Taiwan; ^4^ Department of Medical Research China Medical University Hospital Taichung Taiwan; ^5^ Division of Medical Genetics China Medical University Children's Hospital Taichung Taiwan; ^6^ Department of Biotechnology and Bioinformatics Asia University Taichung Taiwan; ^7^ Department of Psychiatry China Medical University Hospital Taichung Taiwan; ^8^ College of Medicine China Medical University Taichung Taiwan

**Keywords:** anxiety, depression, ICU, mortality and readmissions, PTSD

## Abstract

**Purpose:**

Intensive care unit (ICU) mortality has decreased, highlighting improved patient outcomes. However, other critical factors affect post‐ICU survival, including lasting physical, cognitive, and psychological challenges termed postintensive care syndrome (PICS), encompassing depression, anxiety, and posttraumatic stress disorder (PTSD). The prevalence of these conditions among ICU survivors is high and potentially linked to ICU treatment. This study aims to understand these factors using Taiwan's National Health Insurance Research Database (NHIRD), exploring their impact on mortality and readmission rates post‐ICU discharge.

**Methods:**

The National Health Insurance (NHI) program in Taiwan, implemented in 1995, provides healthcare for nearly all residents. Its research arm, NHIRD, contains comprehensive medical data for nationwide studies. This research focuses on ICU patients with specific conditions from 2010 to 2018, using ICD codes for diagnosis. Statistical analyses include Cox proportional hazard models and logistic regression, aiming to assess the incidence and risks of anxiety/depression/PTSD.

**Results:**

ICU patients showed a higher risk of anxiety/depression/PTSD compared to non‐ICU patients (adjusted HR = 1.17), with similar trends for anxiety and depression. Females, younger patients, those with higher CCI scores, on mechanical ventilators, and more extended hospital stays had increased odds of anxiety/depression/PTSD. ICU patients with anxiety/depression/PTSD faced increased risks of death and re‐admission, especially among older males with comorbidities.

**Conclusion:**

This study discovered higher anxiety and depression rates post‐ICU compared to general ward patients, particularly among younger individuals, females, and those with longer hospital stays. Factors such as higher comorbidity scores and mechanical ventilation use were linked to lower odds. Addressing mental health postdischarge is crucial, especially for at‐risk groups.

## Graphical abstract

To understand post‐intensive care syndrome (PICS) using Taiwan's National Health Insurance Research Database (NHIRD).

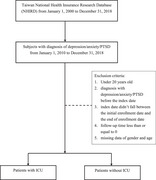

**Abbreviations**
aOR: adjusted odds ratio
CCI: Charlson comorbidity index
CIs: confidence intervals
HRs: hazard ratios
ICD‐9‐CM: International Classification of Diseases, 9th edition, Clinical Modification
ICD‐10‐CM: International Classification of Diseases, 10th edition, Clinical Modification
MV: mechanical ventilator
NHI: National Health Insurance
NHIRD: National Health Insurance Research Database
PICS: postintensive care syndrome
PTSD: posttraumatic stress disorder
SD: standard deviation

## 1 Introduction

Mortality among intensive care unit (ICU) patients also decreased, and this decrease became more pronounced over time (Lai et al. 2018; Zimmerman et al. 2012). Reduced ICU mortality is not the only factor affecting outcomes for critically ill patients (Needham et al. 2012). The physical and mental health and long‐term outcomes of post‐ICU survivors are also important. People who survive critical illness often experience lasting physical, cognitive, and psychological challenges, usually called postintensive care syndrome (PICS) (Needham et al. 2012; Jensen et al. 2015; Brown et al. 2019).

PICS includes mental illnesses such as depression, anxiety disorders, and posttraumatic stress disorder (PTSD) (Prince et al. 2018). Depression can be a severe psychological consequence for patients admitted to the ICU and is frequently seen in survivors of critical illness receiving ICU care (Choi et al. 2016). Among critically ill survivors in 26 ICUs in the United Kingdom, the prevalence of depression is as high as 40% (Hatch et al. 2018). Previous systematic reviews have shown that significant PTSD symptoms are present in survivors of severe illness, ranging from 5% to 63%, and that these symptoms are consistently associated with lower health‐related quality of life (Jackson et al. 2007; Davydow, Gifford, et al. 2008; Wade et al. 2013; Griffiths et al. 2007). In particular, there is a potential elevation of around 30% in symptoms of depression, 40% in anxiety, and 60% in PTSD among ICU survivors. ICU treatment might be linked to the emergence of psychological disorders and associated complications (Peris et al. 2011).

The trends in most of these studies suggest that survivors who experienced ICU are at significantly increased risk for psychiatric disorders compared with the nonhospitalized general population rather than relative to other hospitalized cohorts (Sareen et al. 2020). A study in Taiwan also found that the proportion of patients discharged from ICU with mental illness was higher than that of the general population. This result was obtained by comparing patients admitted to ICU from 2000 to 2015 with matched nonhospitalized groups (Peng et al. 2022). Unfortunately, the relationship between emerging depression, anxiety, and PTSD in survivors compared with non‐ICU admissions has not been confirmed, particularly at 1‐year follow‐up after ICU discharge. Prior study methods may not have entirely excluded underlying health conditions, as individuals who were not hospitalized were compared. Depression and anxiety are recognized as factors that elevate the risk of higher mortality rates in the general population (Gilman et al. 2017; Meier et al. 2016). There were limited studies addressing the relationship between depression, anxiety, and readmission rates. One study indicated that prior depression was linked to a higher risk of hospital readmission, whereas past anxiety did not show such an association (Coppler et al. 2023).

Hence, our research aimed to explore the factors linked to depression, anxiety disorders, and PTSD among survivors of ICU treatment, precisely 12 months after their ICU admission (referred to as post‐ICU). We utilized the National Health Insurance Research Database (NHIRD) of Taiwan to assess and compare the risk of depression, anxiety, and PTSD in individuals admitted to the ICU versus those admitted to the general ward. Furthermore, our other objective is to investigate the connection between post‐ICU mental disorders (including depression, anxiety, and PTSD) and both mortality and readmission rates.

## 2 Material and Methods

### 2.1 Data Source

In 1995, the National Health Insurance (NHI) program was implemented in Taiwan to provide healthcare to nearly all residents. It aimed to improve the national health and medical care of Taiwan. The NHIRD, which contains data on the insured person's diagnosis of diseases, prescriptions, and inpatient and outpatient visits, is periodically created for nationwide research and provides general demographic and medical data in an encrypted format. The disease code in this database was based on the International Classification of Diseases, 9th and 10th editions, Clinical Modification (ICD‐9‐CM and ICD‐10‐CM). The NHIRD encrypts patient information to protect their privacy and provides researchers with anonymous identification numbers associated with relevant information, including sex, date of birth, medical services received, and prescriptions. Therefore, patient consent is not required to access the NHIRD. The authors assert that all procedures contributing to this work comply with the ethical standards of the relevant national and institutional committees on human experimentation and with the Helsinki Declaration of 1975, as revised in 2008. This retrospective observational study was approved by the Research Ethics Committee of China Medical University Hospital, Taiwan (CMUH110‐REC1‐038(CR‐3)).

### 2.2 Study Population

In this study, the case (ICU) cohort consists of patients who were admitted to the ICU during any hospitalization with chronic rheumatic heart disease (ICD‐9‐CM code: 393–398; ICD‐10‐CM code: I05‐I09) or hypertension (ICD‐9‐CM code: 401–405; ICD‐10‐CM code: I10‐15, N26.2) or ischemic heart disease (ICD‐9‐CM code: 410–414; ICD‐10‐CM code: I20‐25) or disease of pulmonary circulation (ICD‐9‐CM code: 415–417; ICD‐10‐CM code: I26‐28, T80.0XXA,T81.718A, T81.72XA, T82.817A, T82.818A) or other forms of heart disease (including arrhythmias, ICD‐9‐CM code: 420–429; ICD‐10‐CM code: I23,I25,I30‐39,I40‐52, I97,R00.1) or diseases of the arteries, arterioles (ICD‐9‐CM code: 440–448; ICD‐10‐CM code I70‐79) or diseases of veins and lymphatic vessels, as well as other circulatory system diseases, and capillaries (ICD‐9‐CM code: 451–459; ICD‐10‐CM code I80‐89, I95, I97.2,I99.8,I99.9,K64,R58) or respiratory diseases (ICD‐9‐CM code: 460–519; ICD‐10‐CM code: J00‐99, M34.81, R09.1, R09.81, R09.82, R91.1, A22.1, A37.91, A48.1, B25.0, B44.0, B44.81) within 2010 and 2018. Those who didn't require admission to the ICU during hospitalization for the above conditions (non‐ICU) were matched to the case cohort by sex, age (in 5‐year intervals), the year of the index date, and measured confounders to find the control cohort. Propensity score matching was used in a 1:1 ratio. The index date of ICU patients was defined as the first date of ICU admission, and that of the controls was defined as the first date of admission. The end date of the follow‐up of ICU patients was defined as the date of the earliest date of the first occurrence of depression/anxiety/PTSD, and that of the controls was defined as death, emigration, or on December 31, 2018, or one year after the index date, whichever occurred first.

The exclusion criteria of this study included patients under 20 years old, diagnosed with depression/anxiety/PTSD before the index date, the index date didn't fall between the initial enrollment date and the end of enrollment date, the follow‐up time less than or equal to 0, and with missing data of gender and age.

### 2.3 Main Outcome and Covariates

Depression/anxiety/PTSD (depression: ICD‐9‐CM code 296.2, 296.3, 300.4, 309.0, 309.1, 311, ICD‐10‐CM code F32.0‐F32.5, F32.9, F33, F34.1, F43.21/Anxiety: ICD‐9‐CM code 300.0, ICD‐10‐CM code F41/PTSD: ICD‐9‐CM code 309.81, 309.1, ICD‐10‐CM code F43.1) was the primary outcome in our research, and we will also investigate the mentioned diseases separately. If the earliest date of the first occurrence of depression/anxiety/PTSD was between the index date and one year after the index date, we then considered this as having the outcome.

Covariates included age, sex, some related comorbidities, mechanical ventilation (MV), Charlson Comorbidity Index (CCI) score, and length of hospital stay. Comorbidities were as follows: diabetes (ICD‐9‐CM code 250) (ICD_10‐CM code E08‐E13), liver cirrhosis (ICD‐9‐CM code 571) (ICD_10‐CM code K70, K73, K74), COPD (ICD‐9‐CM code 490, 492, 496) (ICD_10‐CM code J41, J42, J43, J44), asthma (ICD‐9‐CM code 493) (ICD_10‐CM code J45), tuberculosis (ICD‐9‐CM code 011, 012, 018) (ICD_10‐CM code A15, A19), AIDS (ICD‐9‐CM code 042, V08) (ICD_10‐CM code B20, Z21), pneumonia (ICD‐9‐CM code 480–487) (ICD_10‐CM code A22.1, A37.91, A48.1, B25.0, B44.0, J10‐J18), stroke (ICD‐9‐CM code 430–438) (ICD_10‐CM code I60‐I69), acute respiratory failure (ICD‐9‐CM code 518.81, 518.82, 518.84) (ICD_10‐CM code J80, J960, J962, J969), septicemia (ICD‐9‐CM code 038, 041.9, 790.7) (ICD_10‐CM code A40, A41, A49.9, B96.89, R65.1, R65.20, R78.81), heart failure (ICD‐9‐CM code 428) (ICD_10‐CM code I50), urinary tract infection (ICD‐9‐CM code 599.0) (ICD_10‐CM code N39.0), shock without documentation of trauma (ICD‐9‐CM code 785.5) (ICD_10‐CM code H49.88, R57, R65.21), autoimmune diseases (ICD‐9‐CM code 710.0‐710.4, 714.0, 714.3) (ICD_10‐CM code M32‐M36), End‐Stage Renal Disease (ICD‐9‐CM code 585) (ICD_10‐CM code N18.5, N18.6), transplantation (ICD‐9‐CM code V42.0, V42.1, V42.6, V42.7, V42.84) (ICD_10‐CM code Z94.0, Z94.1, Z94.2, Z94.4, Z94.82, Z94.83), solid organ cancer (ICD‐9‐CM code 14–18, 191–195) (ICD_10‐CM code C0‐C6, C70‐C76, C7A), hematological cancer (ICD‐9‐CM code 204–208) (ICD_10‐CM code C91‐C95), and ischemic heart diseases (ICD‐9‐CM code 410–414) (ICD_10‐CM code I20‐I25). These comorbidities and MV were defined prior to the end date.

### 2.4 Statistical Analysis

We presented the categorical data of the study participants as numbers and percentages, and the continuous variables were demonstrated as mean and standard deviation (SD). The incidence rate is the number of events divided by person‐years. The relative risks of developing outcomes in relation to the baseline characteristics (i.e., sex, age) were analyzed and presented as hazard ratios (HRs) and 95% confidence intervals (CIs) through univariate and multivariate Cox proportional hazards models. In addition, we used logistic regression to examine the association between depression/anxiety/PTSD and clinical characteristics. Adjusted models were controlled for sex, age, comorbidities, CCI, MV, and hospital day. The Kaplan–Meier survival curve compared the cumulative incidence between case and control cohorts, and the log‐rank test examined the differences. All statistical analyses were presented using software SAS, version 9.4, and plots were plotted by software R, version 4.0. A significant level was *p*‐value less than 0.05.

## 3 Result

### 3.1 Clinical Characteristics in Patients With and Without ICU

From January 1, 2010 to December 31, 2018, there were a total of 488,176 participants enrolled in this study, with 244,088 people in each cohort. The two groups were remarkably balanced in terms of their baseline demographics/measured confounders. Details of demographic characteristics between the two cohorts are presented in Table 1.

**TABLE 1:** Clinical characteristics in patients with and without ICU.

Variable	ICU, *n* (%)		*p* value
	No	Yes	
All	244,088	244,088	
Sex			0.20
Female	85,404 (35.0)	85,830 (35.2)	
Male	158,684 (65.0)	158,258 (64.8)	
Age group (year)			0.00
<50	26,293 (10.8)	29,441 (12.1)	
50–64	62,809 (25.7)	63,693 (26.1)	
≥65	154,986 (63.5)	150,954 (61.8)	
Age (year)^a^	69.2 (0.03)	68.5 (15.6)	<0.001
DM	67,310 (27.6)	67,054 (27.5)	0.41
ESRD	5074 (2.08)	5193 (2.13)	0.24
Liver cirrhosis	55,728 (22.8)	55,906 (22.9)	0.54
COPD	65,803 (27.0)	65,448 (26.8)	0.25
Asthma	28,827 (11.8)	29,029 (11.9)	0.37
Tuberculosis	6686 (2.74)	6755 (2.77)	0.55
AIDS	256 (0.10)	252 (0.10)	0.86
Autoimmune diseases	1695 (0.69)	1720 (0.70)	0.67
Solid organ cancer	40,759 (16.7)	41,816 (17.1)	<0.001
Hematological cancer	554 (0.23)	616 (0.25)	0.07
Transplantation	337 (0.14)	335 (0.14)	0.94
Ischemic heart diseases	105,314 (43.2)	93,933 (38.5)	<0.001
Pneumonia	81,347 (33.3)	82,777 (33.9)	<0.001
Stroke	79,072 (32.4)	77,253 (31.7)	<0.001
Acute respiratory failure	13,476 (5.52)	15,669 (6.42)	<0.001
Septicemia	37,432 (15.3)	38,820 (15.9)	<0.001
Heart failure	36,141 (14.8)	35,997 (14.8)	0.56
Urinary tract infection	80,054 (32.8)	81,429 (33.4)	<0.001
Shock without documentation of trauma	12,415 (5.09)	13,617 (5.58)	<0.001
CCI			<0.001
0	51,941 (21.3)	56,045 (23.0)	
1	77,762 (31.9)	72,535 (29.7)	
2	52,821 (21.6)	52,787 (21.6)	
3+	61,564 (25.2)	62,721 (25.7)	
Mechanical ventilator	34,620 (14.2)	39,487 (16.2)	<0.001
Hospital day group			<0.001
1–3	55,260 (22.6)	58,528 (24.0)	
4–6	62,018 (25.4)	60,258 (24.7)	
7–13	76,762 (31.5)	73,450 (30.1)	
14+	50,048 (20.5)	51,852 (21.2)	
Hospital day	10.2 (25.1)	10.1 (11.1)	0.01
Follow‐up time from ICU to mental disorders onset (year)^a^	0.87 (0.29)	0.82 (0.35)	<0.001

*Note*: Chi‐square test.

^a^ *t*‐test.

### 3.2 Risk of Depression/Anxiety/PTSD Associated With ICU

According to Table 2, we calculated the risk of depression/anxiety/PTSD associated with ICU. A total of 212,281 person‐years and 200,649 person‐years were followed up in the ICU cohort and non‐ICU cohort. It presents that participants in the non‐ICU cohort had a higher risk of depression/anxiety/PTSD (adjusted HR = 1.17, 95%CI = 1.13, 1.21) than in the ICU cohort after adjusted with age, sex, CCI score, and measured confounders. Furthermore, when we look at each outcome separately, we could see that the non‐ICU cohort also had a higher risk of anxiety and depression (anxiety: adjusted HR = 1.11, 95%CI = 1.07, 1.15; depression: adjusted HR = 1.28, 95%CI = 1.22, 1.35). As shown in Figure S1, for the ICU cohort, the cumulative incidence of the outcome, except PTSD, was significantly higher than the non‐ICU cohort.

**TABLE 2:** Risk of depression/anxiety/PTSD associated with ICU.

	ICU cohort			Non‐ICU cohort				
Variable	Event	Person‐years	IR 1000 person‐years	Event	Person‐years	IR^#^ 1000 person‐years	Crude HR (95% CI)	Adjusted HR (95% CI)
Depression/anxiety/PTSD	7727	212,281	36.40	8515	200,649	42.44	1.17 (1.13, 1.21)***	1.17 (1.13, 1.21)***
Depression	2747		12.90	3348		16.69	1.29 (1.23, 1.36)***	1.28 (1.22, 1.35)***
PTSD	41		0.19	44		0.22	1.14 (0.75, 1.75)	1.04 (0.68, 1.59)
Anxiety	5178		24.40	5391		26.90	1.11 (1.06, 1.15)***	1.11 (1.07, 1.15)***

*Note*: IR, incidence rate per 1000 person‐years; Crude HR: relative hazard ratio; Adjusted HR: multivariable analysis including age, sex, CCI score, mechanical ventilator, hospital day, and comorbidities.

***p < 0.001.

### 3.3 Risk of Depression/Anxiety/PTSD Associated With Clinical Characteristics in Post‐ICU Patients

Table 3 illustrates the association between depression/anxiety/PTSD and clinical characteristics in post‐ICU patients. Compared to males, females had higher odds of developing depression/anxiety/PTSD. The adjusted odds ratio (aOR) was 1.33 (95%CI = 1.27, 1.40). Depression/anxiety/PTSD events were associated with increased odds of younger patients when we compared to older patients (<50 years: aOR = 1.18, 95%CI = 1.10, 1.27; 50–64 years: aOR = 1.18, 95%CI = 1.12, 1.25). The association was also significant when looking into the CCI score, when “CCI score equal to zero” was the reference, the aORs were as follows: (1) aOR = 0.89, 95%CI = 0.83, 0.94; (2) aOR = 0.86, 95%CI = 0.80, 0.93; (3) aOR = 0.81, 95%CI = 0.75, 0.88. In the same way, patients using MV were 0.77 times (95%CI = 0.72, 0.83) more likely to have depression/anxiety/PTSD than people without taking MVs. Furthermore, patients with longer hospital stays had higher odds of developing depression/anxiety/PTSD compared to patients with shorter hospital stays (4–6 days: aOR = 1.10, 95%CI = 1.03, 1.17; 7–13 days: aOR = 1.30, 95%CI = 1.22, 1.39; ≥14 days: aOR = 1.67, 95%CI = 1.55, 1.80).

**TABLE 3:** Risk of depression/anxiety/PTSD associated with clinical characteristics in post‐ICU patients.

	depression/anxiety/PTSD, *n* (%)			
Variable	No	Yes	Crude OR (95% CI)	Adjusted OR (95% CI)
Sex				
Female	82,367 (35.0)	3463 (40.7)	1.28 (1.22, 1.33)***	1.33 (1.27, 1.40)***
Male	153,206 (65.0)	5052 (59.3)	1 (Reference)	1 (Reference)
Age group (year)				
<50	28,324 (12.0)	1117 (13.1)	1.16 (1.08, 1.23)***	1.18 (1.10, 1.27)***
50–64	61,281 (26.0)	2412 (28.3)	1.15 (1.10, 1.21)***	1.18 (1.12, 1.25)***
≥65	145,968 (62.0)	4986 (58.6)	1 (Reference)	1 (Reference)
Comorbidity				
DM	65,106 (27.6)	1948 (22.9)	0.78 (0.74, 0.82)***	0.80 (0.76, 0.85)***
ESRD	5026 (2.13)	167 (1.96)	0.92 (0.79, 1.07)	1.07 (0.91, 1.26)
Liver cirrhosis	53,900 (22.9)	2006 (23.6)	1.04 (0.99, 1.09)	1.12 (1.06,1.18)***
COPD	63,232 (26.8)	2216 (26.0)	0.96 (0.91, 1.01)	1.07 (1.01, 1.13)***
Asthma	28,932 (11.9)	1097 (12.9)	1.10 (1.03, 1.17)**	1.16 (1.08, 1.24)***
Tuberculosis	6573 (2.79)	182 (2.14)	0.76 (0.66, 0.88)***	0.89 (0.76, 1.03)
AIDS	244 (0.10)	8 (0.09)	0.91 (0.45, 1.84)	1.11 (0.55, 2.26)
Autoimmune diseases	1666 (0.71)	54 (0.63)	0.90 (0.68, 1.18)	0.86 (0.65, 1.13)
Solid organ cancer	40,660 (17.3)	1156 (13.6)	0.75 (0.71, 0.80)***	0.84 (0.78, 0.91)***
Hematological cancer	600 (0.25)	16 (0.19)	0.74 (0.45, 1.21)	0.90 (0.54, 1.48)
Transplantation	326 (0.14)	9 (0.11)	0.76 (0.39, 1.48)	0.79 (0.40, 1.54)
Ischemic heart diseases	90,586 (38.5)	3347 (39.3)	1.04 (0.99, 1.08)	1.15 (1.09, 1.21)***
Pneumonia	80,232 (34.1)	2545 (29.9)	0.83 (0.79, 0.87)***	0.90 (0.86, 0.95)***
Stroke	74,014 (31.4)	3239 (38.0)	1.34 (1.28, 1.40)***	1.36 (1.29, 1.42)***
Acute respiratory failure	15,461 (6.56)	208 (2.44)	0.36 (0.31, 0.41)***	0.46 (0.40, 0.54)***
Septicemia	37,936 (16.1)	884 (10.4)	0.60 (0.56, 0.65)***	0.69 (0.64, 0.75)***
Heart failure	34,877 (14.8)	1120 (13.2)	0.87 (0.82, 0.93)***	0.90 (0.84, 0.96)**
Urinary tract infection	78,621 (33.4)	2808 (33.0)	0.98 (0.94, 1.03)	0.99 (0.94, 1.04)
Shock without documentation of trauma	13,352 (5.67)	265 (3.11)	0.54 (0.47, 0.61)***	0.74 (0.65, 0.84)***
CCI				
0	53,864 (22.9)	2181 (25.6)	1 (Reference)	1 (Reference)
1	69,841 (29.7)	2694 (31.6)	0.95 (0.90, 1.01)	0.89 (0.83, 0.94)***
2	51,037 (21.7)	1750 (20.6)	0.85 (0.79, 0.90)***	0.86 (0.80, 0.93)***
3+	60,831 (25.8)	1890 (22.2)	0.77 (0.72, 0.82)***	0.81 (0.75, 0.88)***
Mechanical ventilator	38,438 (16.3)	1049 (12.3)	0.72 (0.68, 0.77)***	0.77 (0.72, 0.83)***
Hospital day				
1–3	56,788 (24.1)	1740 (20.4)	1 (Reference)	1 (Reference)
4–6	58,293 (24.8)	1965 (23.1)	1.10 (1.03, 1.18)**	1.10 (1.03, 1.17)**
7–13	70,773 (30.0)	2677 (31.4)	1.23 (1.16, 1.31)***	1.30 (1.22, 1.39)***
14+	49,719 (21.1)	2133 (25.1)	1.40 (1.31, 1.49)***	1.67 (1.55, 1.80)***

*Note*: Crude OR: relative odds ratio; Adjusted OR: multivariable analysis including age, sex, CCI score, mechanical ventilator, hospital day, and comorbidities.

**p < 0.01, ***p < 0.001.

### 3.4 Risk of Death Associated With Depression/Anxiety/PTSD and Clinical Characteristics in Post‐ICU Patients

As Table 4 demonstrates, a total of 50,723 patients died after ICU. Patients with depression/anxiety/PTSD had a higher risk of death than patients without (adjusted HR = 1.07, 95%CI = 1.03, 1.11). Males had a higher risk of death than females (adjusted HR = 1.12, 95%CI = 1.03, 1.11). Also, the older the age, the higher the risk of death (50–64: adjusted HR = 1.43, 95%CI = 1.36, 1.50; ≥65: adjusted HR = 3.82, 95%CI = 3.65, 3.99). Besides, among patients diagnosed with comorbidities like heart failure, stroke, and COPD, the risk of death was higher than those without being diagnosed with comorbidities. Furthermore, as the CCI score increased or the length of hospital stay increased, the higher the risk of death.

**TABLE 4:** Risk of death associated with depression/anxiety/PTSD and clinical characteristics in post‐ICU patients.

Variable	Event *N* = 50,723	Person‐years	IR 100 person‐years	Crude HR (95% CI)	Adjusted HR (95% CI)
Depression/anxiety/PTSD					
No	47,959	714,615	67.10	1 (Reference)	1 (Reference)
Yes	2764	37,994	72.80	1.09 (1.05, 1.13)***	1.07 (1.03, 1.11)***
Sex					
Female	19,447	256,624	75.80	1 (Reference)	1 (Reference)
Male	31,276	495,986	63.10	0.83 (0.82, 0.85)***	1.12 (1.10, 1.14)***
Age group (year)					
<50	2197	118,714	18.50	1 (Reference)	1 (Reference)
50–64	7161	238,268	30.10	1.63 (1.55, 1.71)***	1.43 (1.36, 1.50)***
≥65	41,365	395,627	104.60	5.67 (5.43, 5.92)***	3.82 (3.65, 3.99)***
DM	17,163	202,894	84.60	1.38 (1.36, 1.41)***	0.94 (0.92, 0.96)***
ESRD	2003	12,893	155.40	2.35 (2.25, 4.46)***	1.48 (1.41, 1.55)***
Liver cirrhosis	11,385	158,998	71.60	1.08 (1.06, 1.10)***	1.01 (0.99, 1.03)
COPD	17,722	164,726	107.60	1.91 (1.88, 1.95)***	1.21 (1.19, 1.23)***
Asthma	7198	81,713	88.10	1.36 (1.32, 1.39)***	0.86 (0.83, 0.88)***
Tuberculosis	1799	14,757	121.90	1.83 (1.75, 1.92)***	1.18 (1.13, 1.24)***
AIDS	26	693	37.50	0.56 (0.38, 0.82)***	0.53 (0.36, 0.79)**
Autoimmune diseases	407	4911	82.90	1.23 (1.12, 1.36)***	0.99 (0.90, 1.09)
Solid organ cancer	11,062	81,445	135.80	2.29 (2.25, 2.34)***	1.27 (1.24, 1.30)***
Hematological cancer	121	931	130.00	1.93 (1.61, 2.30)***	1.51 (1.26, 1.80)***
Transplantation	46	1193	38.60	0.58 (0.43, 0.77)***	0.70 (0.10, 5.03)
Ischemic heart diseases	18,760	337,049	55.70	0.73 (0.71, 0.74)***	0.82 (0.80, 0.83)***
Pneumonia	20,615	192,024	107.40	2.00 (1.96, 2.03)***	1.38 (1.36, 1.41)***
Stroke	20,382	222,371	91.70	1.60 (1.57, 1.63)***	1.10 (1.08, 1.12)***
Acute respiratory failure	1795	13,606	131.90	1.99 (1.90, 2.08)***	1.43 (1.36, 1.50)***
Septicemia	9929	70,834	140.17	2.34 (2.29, 2.39)***	1.42 (1.38, 1.45)***
Heart failure	11,599	93,368	124.20	2.09 (2.05, 2.13)***	1.53 (1.50, 1.56)***
Urinary tract infection	21,195	201,971	104.90	1.96 (1.92, 1.99)***	1.27 (1.24, 1.29)***
**Shock without documentation of trauma**					
CCI					
0	6598	204,143	32.30	1 (Reference)	1 (Reference)
1	12,565	260,113	48.30	1.50 (1.45, 1.54)***	1.36 (1.32, 1.40)***
2	12,594	156,618	80.40	2.49 (2.42, 2.57)***	1.82 (1.76, 1.88)***
3+	18,966	131,736	144.00	4.48 (4.35, 4.60)***	2.63 (2.54, 2.73)***
Mechanical ventilator	6744	87,088	77.40	1.17 (1.14, 1.20)***	1.00 (0.97, 1.03)
Hospital days					
1–3	7061	188,931	37.37	1 (Reference)	1 (Reference)
4–6	11,696	202,232	57.83	1.55 (1.50, 1.59)***	1.24 (1.21, 1.28)***
7–13	18,096	228,212	79.30	2.12 (2.06, 2.18)***	1.44 (1.39, 1.48)***
14+	13,870	133,233	104.10	2.78 (2.70, 2.86)***	1.65 (1.59, 1.70)***

*Note*: IR, incidence rate per 1000 person‐years; Crude HR: relative hazard ratio; Adjusted HR: multivariable analysis including age, sex, CCI score, mechanical ventilator, hospital day, and comorbidities.

**p < 0.01, ***p < 0.001.

### 3.5 Risk of Re‐Admission Associated With Depression/Anxiety/PTSD and Clinical Characteristics in Post‐ICU Patients

In Table 5, a total of 117,631 patients were re‐admitted after ICU. Patients with depression/anxiety/PTSD had a higher risk of re‐admission than patients without (adjusted HR = 1.40, 95%CI = 1.36, 1.43). Males had a higher risk of re‐admission than females (adjusted HR = 1.09, 95%CI = 1.07, 1.10). Also, the older the age, the higher the risk of re‐admission (50–64: adjusted HR = 1.15, 95%CI = 1.13, 1.18; ≥65: adjusted HR = 1.62, 95%CI = 1.59, 1.65). Among patients diagnosed with comorbidities like heart failure, stroke, and COPD, the risk of re‐admission was higher than without diagnosed with comorbidities. Furthermore, as the CCI score increased or the length of hospital stay increased, the higher the risk of re‐admission.

**TABLE 5:** Risk of re‐admission associated with depression/anxiety/PTSD and clinical characteristics in post‐ICU patients.

Variable	Event *N* = 117,631	Person‐years	IR 100 person‐years	Crude HR (95% CI)	Adjusted HR (95% CI)
Depression/anxiety/PTSD					
No	111,367	425,070	26.20	1 (Reference)	1 (Reference)
Yes	6264	17,477	35.80	1.38 (1.35, 1.42)***	1.40 (1.36, 1.43)***
Sex					
Female	41,648	149,371	27.90	1 (Reference)	1 (Reference)
Male	75,983	293,175	25.90	0.94 (0.93, 0.95)***	1.09 (1.07, 1.10)***
Age group (year)					
<50	11,920	79,741	15.00	1 (Reference)	1 (Reference)
50–64	29,636	148,441	20.00	1.30 (1.28, 1.33)***	1.15 (1.13, 1.18)***
≥65	76,075	214,364	35.50	2.17 (2.13, 2.21)***	1.62 (1.59, 1.65)***
DM	36,566	108,061	33.80	1.34 (1.33, 1.36)***	1.04 (1.03, 1.06)***
ESRD	3286	5569	59.00	1.99 (1.92, 2.06)***	1.41 (1.36, 1.47)***
Liver cirrhosis	27,710	88,619	31.30	1.19 (1.17, 1.21)***	1.11 (1.09, 1.12)***
COPD	33,379	86,596	38.60	1.52 (1.50, 1.54)***	1.16 (1.14, 1.17)***
Asthma	15,429	42,869	36.00	1.33 (1.31, 1.36)***	1.02 (1.00, 1.04)*
Tuberculosis	3110	7771	40.00	1.43 (1.38, 1.48)***	1.10 (1.06, 1.14)***
AIDS	88	445	19.80	0.76 (0.61, 0.93)**	0.64 (0.52, 0.79)***
Autoimmune diseases	950	2424	39.20	1.40 (1.31, 1.49)***	1.23 (1.15, 1.31)***
Solid organ cancer	18,833	42,395	44.40	1.68 (1.66, 1.71)***	1.17 (1.15, 1.19)***
Hematological cancer	213	467	45.60	1.66 (1.45, 1.89)***	1.34 (1.17, 1.54)***
Transplantation	212	507	41.80	1.51 (1.32, 1.73)***	1.14 (0.99, 1.31)
Ischemic heart diseases	51,860	189,663	27.30	1.04 (1.03, 1.06)***	1.09 (1.08, 1.11)***
Pneumonia	38,252	104,044	36.80	1.48 (1.46, 1.50)***	1.25 (1.23, 1.27)***
Stroke	39,298	125,242	31.40	1.24 (1.22, 1.25)***	1.06 (1.04, 1.07)***
Acute respiratory failure	2673	7594	35.20	1.29 (1.24, 1.34)***	1.13 (1.09, 1.18)***
Septicemia	16,008	36,001	44.50	1.66 (1.63, 1.69)***	1.30 (1.27, 1.32)***
Heart failure	19,690	47,505	41.50	1.56 (1.54, 1.59)***	1.24 (1.22, 1.26)***
Urinary tract infection	40,166	108,616	37.00	1.49 (1.47, 1.51)***	1.21 (1.19, 1.23)***
Shock without documentation of trauma	4425	13,071	33.90	1.25 (1.21, 1.29)***	1.06 (1.03, 1.10)***
CCI					
0	23,480	133,739	17.60	1 (Reference)	1 (Reference)
1	35,289	159,262	22.20	1.24 (1.22, 1.26)***	1.14 (1.12, 1.16)***
2	27,149	85,941	31.60	1.70 (1.67, 1.73)***	1.38 (1.35, 1.41)***
3+	31,713	63,605	49.90	2.52 (2.47, 2.56)***	1.78 (1.74, 1.82)***
Mechanical ventilator	13,910	52,840	26.30	0.99 (0.98, 1.01)	1.01 (0.99, 1.03)
Hospital days					
1–3	25,454	112,055	22.70	1 (Reference)	1 (Reference)
4–6	30,401	119,404	25.50	1.11 (1.09, 1.13)***	1.02 (1.00, 1.04)*
7–13	37,356	135,391	27.60	1.19 (1.18, 1.21)***	1.05 (1.04, 1.07)***
14+	24,420	75,697	32.30	1.38 (1.36, 1.41)***	1.13 (1.11, 1.16)***

*Note*: IR, incidence rate per 1000 person‐years; Crude HR: relative hazard ratio; Adjusted HR: multivariable analysis including age, sex, CCI score, mechanical ventilator, hospital day, and comorbidities.

*p < 0.05, **p < 0.01, ***p < 0.001.

## 4 Discussion

Our investigation revealed that patients admitted to the ICU had a significantly higher incidence and risk of developing depression and anxiety compared to those with the same conditions treated in general wards. However, it remains uncertain whether the observed effects are due to ICU admission itself or the underlying medical condition that necessitated ICU care. To address this, we employed a study design aimed at tackling the issue. Specifically, we compared patients admitted to the ICU with those who had similar medical conditions but were not admitted to the ICU. This approach helps to isolate the effects of different diseases.

Although we cannot eliminate the influence of disease severity, we applied statistical matching methods. Patients who did not require ICU admission during hospitalization for the specified conditions (non‐ICU group) were matched to the ICU cohort based on sex, age (in 5‐year intervals), the year of the index date, and measured confounders. This matching ensured comparability between the ICU and non‐ICU groups in terms of underlying medical conditions and severity. To further account for disease severity, we included variables such as the severity of the underlying medical condition, comorbidities, length of hospital stay, CCI, and MV use in our statistical models. This approach allowed us to control these factors when assessing the independent effect of ICU admission.

In addition, to minimize the potential influence of preexisting psychiatric disorders (e.g., depression, anxiety, or posttraumatic stress disorder), we excluded patients with these conditions prior to the index date. This enabled us to conduct a longitudinal follow‐up study to monitor changes in psychiatric symptoms over time following ICU admission. Our results reflect experiencing a sudden or severe instance of a potentially life‐threatening illness, coupled with potentially traumatic experiences in the ICU, may contribute to the development of treatable psychological problems, such as posttraumatic stress, anxiety, and depression (Girard et al. 2007; Desai et al. 2011). Although our findings are consistent with the increased susceptibility to depression and anxiety observed in ICU survivors, our findings do not suggest a higher incidence of PTSD. Furthermore, research shows that PTSD often coexists with other conditions, such as depression and various anxiety disorders (Brady et al. 2000). Our study suggests potential underdiagnosis of PTSD due to symptom overlap with depression and anxiety disorders. Diagnosis challenges arise from compatible criteria use, leading to missed PTSD cases when trauma history is not explored. In addition to PTSD, mental disorders might be undiagnosed, either. Therefore, long‐term psychological sequelae and interventions are necessary.

Previous studies indicate that younger adults and females in ICU have a higher susceptibility to mental disorders (Desai et al. 2011). In Taiwan, our research highlighted younger age and female gender as notable risk factors for depression, anxiety, and PTSD among ICU patients. Female gender, specifically, showed an independent link with increased psychological distress levels (McKinley et al. 2012). Several theories have been suggested to explain this age‐related finding, with many highlighting the overlooked risk of depression in older adults (Kessler et al. 2010). Younger individuals often have better survival rates and easier access to mental health services. However, they face unique challenges like academic pressures, uncertain careers, and complex relationships, leading to higher stress and depression. Further research is needed to uncover additional contributing factors.

Chronic medical illness is associated with depression and anxiety disorders (Fiest et al. 2011). In our findings, we found that ICU patients with liver cirrhosis, chronic obstructive pulmonary disease, asthma, ischemia heart disease, and stroke were at higher risk of developing depression, anxiety, and PTSD, and the findings align with the survey among those patients (Hernaez et al. 2022; Long et al. 2020; Plank et al. 2023; Johnson et al. 2020; Chun et al. 2022). However, conditions like diabetes, solid organ tumors, pneumonia, sepsis, heart failure, and shock are associated with a lower risk of developing these disorders, prompting the need for more investigation.

Our research found that a higher CCI was associated with a lower likelihood of developing depression, anxiety, or PTSD after ICU treatment. Patients with more comorbidities were less prone to these disorders post‐ICU. However, our study didn't include patients with pre‐existing mental disorders, which may have influenced our results and requires consideration.

The need for MV could be identified as a vulnerability factor contributing to the occurrence of mental disorders. A study offers crucial insights into the prevalence of psychiatric disorders in patients needing MV during critical illness, along with the risks associated with psychiatric diagnoses and psychiatric medication use post‐ICU discharge (Wunsch et al. 2014). However, our findings were not congruent with the above report. The factors contributing to this phenomenon may include a heightened probability of mortality and complications (He et al. 2021). In cases involving severe physical conditions, there is a tendency to overlook underlying mental disorders.

While some studies have not discovered a correlation between the duration of hospital or ICU stay and an elevated risk of anxiety and depression (Nikayin et al. 2016; Battle et al. 2015), it is essential to note the variability in findings across different research investigations. Studies of survivors of acute lung injury or acute respiratory distress syndrome have identified prolonged ICU length of stay prolonged sedation, and prolonged MV as potential risk factors for post‐ICU psychopathology (Davydow, Desai, et al. 2008). While our data did not include information on ICU length of stay, our study found that longer overall hospitalization was associated with an increased risk of developing mental disorders. Further clinical research is needed to identify potential contributing factors.

Our study discovered an association between post‐ICU depression, anxiety, and PTSD and increased mortality for 12 months following ICU discharge. The association persists when adjusted for age, sex, illness severity (CCI), the length of hospital stays, and the presence of other psychopathological issues. A large UK study also showed depression had higher mortality following ICU discharge (Hatch et al. 2018). For comparison, a large US study in a general veterans’ population estimates that diagnosed depression is associated with a 17% greater hazard of all‐cause mortality at 3 years (Zivin et al. 2015).

The link between depression, anxiety, PTSD, and mortality among post‐ICU individuals may be influenced by chronic illness severity and pre‐ and postdischarge factors not addressed here. Detecting and managing depression symptoms post‐ICU discharge is crucial, highlighting the need for vigilance in ICU follow‐up care. Moreover, these mental health issues also heightened readmission risks.

### 4.1 Strengths and Limitations

This study's strength lies in its sizable patient sample, representing nationwide ICU admissions for high external validity. Unlike prior studies, it compares ICU patients with non‐ICU controls, accounting for hospitalization effects. We focused on cardiopulmonary disease cases to mitigate confounding factors. Individuals with pre‐existing psychopathological conditions are at risk of exacerbation post‐ICU treatment. A specific phenotype suggests those with depression, anxiety, or PTSD are prone to critical illness (Hatch et al. 2018). We excluded participants with prior psychiatric diagnoses to reduce confounding. This approach enhances the study's credibility and contributes to a more accurate understanding of psychiatric risks in post‐ICU care.

This study has several notable limitations. First, due to inherent confounding factors in LHID claims, detailed critical illness characteristics and relevant data like habits, biomarkers, and psychosocial factors were unavailable, affecting our ability to thoroughly assess ICU admission‐mental disorder risk associations. We lacked data on potential mediators specific to the ICU stay, such as delirium, sedation practices, sleep disruption, or traumatic experiences (e.g., invasive procedures, isolation). In addition, we did not have information on the length of ICU stay and regarding the length of MV. The disease severity at admission (e.g., using APACHE II scores) was also not provided. Furthermore, we were unable to explore biological factors (e.g., systemic inflammation, neurohormonal stress responses) or environmental factors (e.g., ICU trauma, prolonged immobility) to distinguish the physical effects of illness from the psychological impacts of ICU care. Further clinical investigations with comprehensive data collection are needed to clarify this relationship. For example, a subgroup analysis based on ICU stay duration (e.g., < 48 h, 2–7 days, > 7 days) could be conducted to examine whether psychiatric outcomes vary across these groups. In addition, disease severity, MV duration, and other ICU‐related factors (e.g., sedation and use of restraints) should be included in multivariate models. Second, as an observational study using international disease codes, mental disorders might be underdiagnosed, particularly in individuals not seeking medical attention. Lastly, the administrative dataset's diagnosis of mental disorders lacks validation, posing challenges and potentially overrepresenting subclinical cases in Taiwan's health insurance system. Medical expenditure structures, emphasizing diagnosis codes for billing, impact disease recording accuracy (Edwards et al. 2020). Future studies on post‐ICU survivors and psychiatric disorders should address these concerns for a more comprehensive understanding.

## 5 Conclusion

This study noted higher rates of anxiety and depression 12 months post‐ICU compared to general ward patients, especially among younger individuals, females, and those with longer hospital stays. Interestingly, higher CCI and MV use was linked to lower rates of these mental health issues. However, residual confounders require further investigation to clarify these associations. In addition, post‐ICU depression, anxiety, and PTSD were tied to increased mortality and readmission. Clinicians should prioritize mental health support postdischarge, especially for at‐risk groups like women, younger patients, and those with multiple comorbidities.

## Author Contributions

**Chen‐Shu Chang**: writing–original draft, writing–review and editing, conceptualization, methodology. **Fuu‐Jen Tsai**: conceptualization, writing–review and editing, data curation, formal analysis. **Chun‐Hui Liao**: conceptualization, writing–review and editing, writing–original draft, methodology, project administration, supervision.

## Ethics Statement

The authors also thank the Hospital of China Medical University Institutional Review Board for approving the study (CMUH110‐REC1‐038(CR‐3)).

## Conflicts of Interest

The authors declare no conflicts of interest.

### Peer Review

The peer review history for this article is available at https://publons.com/publon/10.1002/brb3.70319

## Supporting information



Supplemental Figure S1 The cumulative incidence of depression, anxiety, and PTSD

## Data Availability

The dataset utilized in this study is maintained by the Taiwan Ministry of Health and Welfare (MOHW). Access to the current data requires approval from the MOHW. Researchers interested in obtaining access to this dataset can submit an application form to the MOHW at the following email address: stcarolwu@mohw.gov.tw. The Taiwan Ministry of Health and Welfare is located at No.488, Sec. 6, Zhongxiao E. Rd., Nangang Dist., Taipei City 115, Taiwan (R.O.C.). For inquiries, please contact them via phone at +886‐2‐8590‐6848. All pertinent data are included in the paper.
