# Supplementary figures and images for: Associations Between Elevated Rates of Depression, Anxiety, and PTSD Among ICU Survivors and Increased Mortality and Readmissions
Source: Brain Behav. 2025 Feb 16;15(2):e70319. doi: 10.1002/brb3.70319 (PMC11830751; doi:10.1002/brb3.70319)

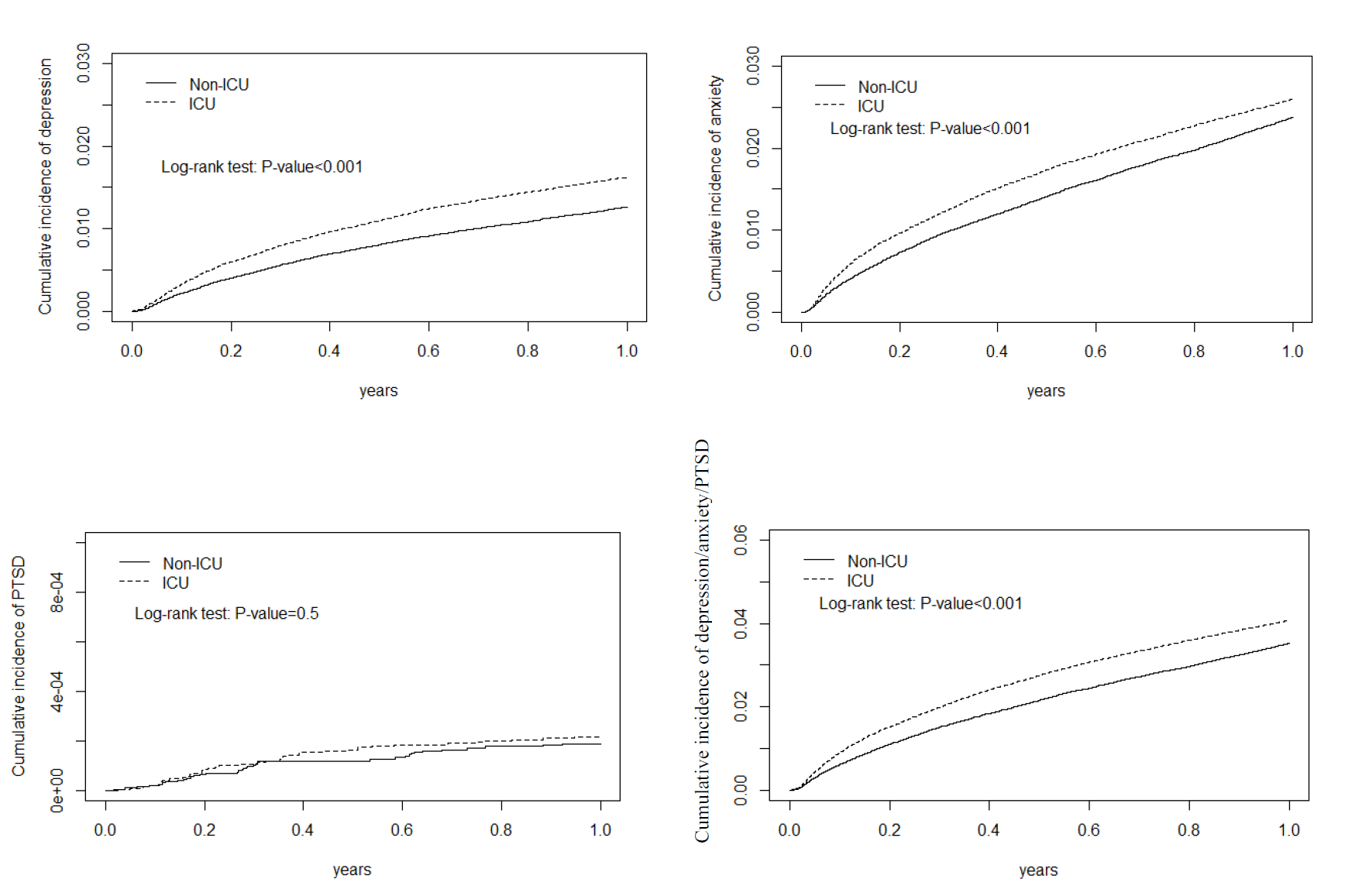

Supplement: Supplementary file 1 — Supplemental Figure S1 The cumulative incidence of depression, anxiety, and PTSD [file BRB3-15-e70319-s001.tif]
